# The Role of B Cells and B Cell Therapies in Immune-Mediated Liver Diseases

**DOI:** 10.3389/fimmu.2021.661196

**Published:** 2021-04-14

**Authors:** Tamsin Cargill, Emma L. Culver

**Affiliations:** ^1^ Peter Medawar Building for Pathogen Research, Nuffield Department of Medicine, University of Oxford, Oxford, United Kingdom; ^2^ Oxford Liver Unit, John Radcliffe Hospital, Oxford, United Kingdom

**Keywords:** IgG4-related disease, autoimmune hepatitis, primary biliary cholangitis (PBC), primary sclerosing cholangites (PSC), B cell, Rituximab

## Abstract

B cells form a branch of the adaptive immune system, essential for the body’s immune defense against pathogens. B cell dysfunction has been implicated in the pathogenesis of immune mediated liver diseases including autoimmune hepatitis, IgG4-related hepatobiliary disease, primary biliary cholangitis and primary sclerosing cholangitis. B cells may initiate and maintain immune related liver diseases in several ways including the production of autoantibodies and the activation of T cells via antigen presentation or cytokine production. Here we comprehensively review current knowledge on B cell mechanisms in immune mediated liver diseases, exploring disease pathogenesis, B cell therapies, and novel treatment targets. We identify key areas where future research should focus to enable the development of targeted B cell therapies.

## Introduction

B cells form a branch of the adaptive immune system that confer long-lived targeted responses to pathogens and other non–self-proteins (1). Developing in the bone marrow and spleen, B cells mature from lymphoid progenitors to mature B cells which express B cell receptors, each with its own unique antigen affinity. On meeting its cognate antigen, naïve B cells proliferate within B cell follicles producing short lived antigen secreting cells and germinal center (GC) B cells. GC B cells undergo rounds of proliferation and somatic hypermutation within GCs, resulting in populations of cells including long-lived memory B cells, plasmablasts (PB) and plasma cells (PC), which are able to secrete high affinity antibodies (1). Antibody subclass is directed by class switch recombination, which enables the production of several immunoglobulin (Ig) subtypes IgM, IgD, IgG (types 1-4), IgA and IgE [reviewed in (2)]. B cells can affect immune control directly via neutralizing antibodies, or indirectly via communication with the complement cascade and other effector immune cells such as macrophages and T cells through cytokine production or antigen presentation [reviewed in (3)]. B cells can also have regulatory functions, through production of anti-inflammatory cytokines [reviewed in (4)].

B cell dysfunction has been implicated in the pathogenesis of a number of immune mediated liver diseases including autoimmune hepatitis (AIH), IgG4-related hepatobiliary disease (IgG4-HBD), primary biliary cholangitis (PBC) and primary sclerosing cholangitis (PSC). Here we review current knowledge on B cell mechanisms in immune mediated liver diseases, exploring disease pathogenesis, B cell therapies, and novel treatment targets. The role of B cells in biliary atresia and drug-induced liver injury has been explored elsewhere (5) and will not be covered in this review.

## Autoimmune Hepatitis

Autoimmune hepatitis (AIH) is a chronic inflammatory liver disease characterized by raised serum total immunoglobulin G (IgG), the presence of circulating autoantibodies and liver histology showing interface hepatitis with a lymphoplasmacytic infiltrate (6). Although no single disease trigger has been identified, multiple genetic, epigenetic and environmental factors are associated with AIH development [reviewed in (7, 8)]. Corticosteroids induce disease remission and immunosuppressives such as thiopurines and mycophenolate are used to maintain the therapeutic response. Biochemical and histological disease relapse is frequent on withdrawal of immunosuppression.

Whilst early studies supported T cell dysregulation as being central to AIH pathogenesis [reviewed in (8)], a role for B cells became evident when B cell depletion with Rituximab was shown to induce clinical improvement in AIH patients refractory to conventional therapy (9, 10) and in murine models of AIH (11). Several pathogenic mechanisms of B cells in AIH have been proposed including the generation of auto-reactive antibodies, B cell overactivation, excess immunoglobulin production and the recruitment of T cells through cytokine production and antigen presentation.

### Auto-Antibodies in AIH

Specific autoreactive antibodies can be detected in AIH patients, including anti-nuclear (ANA), anti-smooth muscle (SMA), anti-liver-kidney microsomal type 1 or 3 (anti-LKM-1, anti-LKM-3), anti-liver cytosol type 1 (anti-LC1), anti-soluble liver antigen (anti-SLA) or anti-asialoglycoprotein receptor (ASGPR) [reviewed in (12)]. Total IgG levels and titers of autoantibodies are part of the diagnostic criteria for AIH and can correlate with liver biochemical and histological markers of disease activity (6, 13). Autoantibody detection can also be used to sub-classify AIH into type 1 and type 2, the latter affecting younger individuals with increased disease severity (**Table 1**).

**Table 1:** Autoantibodies in AIH.

AIH subtype	Associated auto-antibodies
**AIH type 1**	Anti-nuclear antibodies (ANAs) Anti-smooth muscle antibodies (SMA) Anti-neutrophil cytoplasmic antibodies (ANCA) Anti-asialoglycoprotein receptor (ASGPR) Anti-soluble liver (anti-SLA)
**AIH type 2**	Anti-liver kidney microsomal antigens type 1 or type 3 (anti-LKM-1, anti-LKM-3) Anti-liver cytosol type 1 (anti-LC1)

The events leading to autoantibody production in AIH are not clear, but some evidence suggests they might form in response to infection with hepatitis C virus or human herpes virus 6, where viral and human epitopes share sequence homology (14, 15). Although autoantibody detection has a predominantly diagnostic role, there is evidence that anti-LKM-1 and anti-LC1 are directly pathogenic. Anti-LKM-1 production is triggered by expression of cytochrome P450 2D6 (CYP2D6) on the surface of hepatocytes in AIH. It may be directly cytotoxic or activate auto-reactive T cells that target CYP2D6 expressing hepatocytes (14).

### Activation of B Cells and IgG Production

Activated B cells may be a determinant of disease activity in AIH. In new onset AIH there are increased numbers of activated B cells and PC compared to healthy controls (16). PCs positively correlate with serum IgG levels, suggesting they might be the source of excess IgG production, characteristic of active disease (16).

Several factors might regulate B cell overactivation in AIH. Circulating follicular helper T (Tfh) cells necessary for B cell differentiation and maturation and their hallmark cytokine IL-21, which drives B cell activation, PC differentiation, and immunoglobulin production, are increased in AIH and correlate with serum IgG and hepatic inflammation (16, 17). Blockade of IL-21 suppresses Tfh cell generation and can prevent AIH development in murine models. B cell activating factor (BAFF), necessary for B cell survival and differentiation, is also increased in the serum of individuals with AIH (18, 19). BAFF levels positively correlate with liver transaminases and histological liver inflammation, but not with serum IgG titer (19).

Finally, T cells themselves might be the drivers of B cell activation in AIH. Sequencing of B and T cell receptors in the blood and liver of individuals with AIH showed skewing of T rather than B cell receptor profiles (20). T cell expression of activation markers PD1 and CD38 and the magnitude of *ex-vivo* cytokine responses towards autoantigenic peptides correlates with AIH disease activity (20–22). Autoreactive T cell responses specific to autoantigen SLA have been investigated by flow cytometry and single cell RNA sequencing (23), showing SLA CD4 T cells transcriptionally upregulate expression of genes associated with B cell help and inflammation. These cells are similar in profile to so called T peripheral helper (Tph) cells that have been identified as drivers of B cell inflammation in other autoimmune conditions such as Rheumatoid Arthritis and IgG4-related disease (24). The population of PD1 positive activated Tph cells was expanded amongst all CD4 T cells in AIH, produced IL-21 and interferon gamma (IFN*γ*) on ex-vivo restimulation and drove B cell differentiation in co-cultures (23).

Together these data suggest overactivation of the B cell axis in AIH is associated with IgG production and liver inflammation.

### Antigen Presentation in AIH

Antigen presentation by B cells to T cells may play an important role in AIH pathogenesis. In patients with new onset AIH, there is increased expression of CD86 on B cells (16), suggesting they are primed for T cell co-stimulation. In a murine model of AIH, B cell depletion was associated with a reduced cytotoxic and proliferative capacity of intrahepatic T cells, implying in this model at least, B cells are necessary for T cell function (11). *Ex-vivo* proliferation assays of T cells from these AIH mice showed that CD19+ B cells as compared to CD19- lymphocytes were effective antigen presenting cells to CD4+ T cells (11). There is also evidence that B and T cells might share epitopes for the same autoantigens, as LKM1 specific immune responses have overlap between B and T cell epitope sequences (21). Together these observations suggest the B and T cell arms of the adaptive immune response respond to the same autoantigens in AIH, and B cells are necessary to support T cell function through antigen presentation.

### Cytokines Regulating Inflammation and Recruitment in AIH

Cytokine and chemokines provide signals that orchestrate the immune response. In a murine model of AIH, hepatic B cells expressed higher levels of proinflammatory cytokines including interferon gamma (IFN*γ*) than B cells from healthy murine counterparts. These proinflammatory cytokines might attract T cells, which are found co-located with B cells within inflammatory lesions in the liver (11). B cells can also produce C-X-C motif chemokine ligand 10 (CXCL10 or IP-10), which correlates with liver transaminase levels in individuals with AIH (25). After B cell depletion in AIH there is a decrease in proinflammatory cytokines as well as CXCL10 (10, 11), supporting that B cells are an important source of proinflammatory signals during active disease.

The role of B cells in anti-inflammatory cytokine production has also been investigated. In a mouse model of AIH, B cell depletion prior to AIH onset led a more severe AIH phenotype with increased liver inflammation (26). Adoptive transfer of IL-10 dependent B regulatory cells, ameliorated inflammation by inhibition of T cell responses in this model (26). This was in contrast to a different mouse model of AIH, where B cell depletion prevented AIH development (11). These results suggest different B cell subtypes might have different roles in AIH pathogenesis, and that the balance between pro- and anti-inflammatory cytokines might be key.

### The Gut Microbiome in AIH

The liver is anatomically and physiologically linked to the gut microbiota via the enterohepatic circulation (27). The gut microbiota is essential to maintain immune homeostasis of the gut-liver axis and is a major modulator of autoimmunity (27, 28). Shifts in microbiota composition activates a mucosal immune response, causing an imbalance of homeostasis, translocation of bacteria and migration of immune cells into the liver (28). Microbial antigens are recognized by the immune system via presentation on major histocompatibility complex (MHC) class II molecules to CD4+ T cells. Intestinal microbial exposure triggers expansion of B cell populations and antibody production in the liver, with proliferating antibody secreting plasmablasts derived from gut associated lymphoid tissues present in animal and human liver disease (29).

Animal models of AIH provided the first evidence that AIH was associated with reduced genetic diversity of the gut microbiome (30). A recent study in humans confirmed these findings, showing individuals with steroid-naïve AIH have a distinct microbial composition compared with healthy volunteers, with lower intraindividual diversity (31). Expansion of potential pathobiont genus *Veillonella* was associated with active disease status and therefore may be directly involved in AIH pathogenesis (31). Further studies are required to understand whether B cells or other immune mediators are directly involved in the response to the *Veillonella* genus.

## IgG4-Related Hepatobiliary Disease

IgG4-related hepatobiliary disease (IgG4-HBD) is part of the chronic multi-system fibroinflammatory condition IgG4-related disease (IgG4-RD). IgG4-HBD is characterized clinically by masses and/or biliary strictures, elevated serum immunoglobulin G4 (IgG4) subclass levels, and organ infiltration with IgG4-positive plasma cells and CD4 T helper cells, a storiform pattern of fibrosis and obliterative phlebitis (32). IgG4-HBD has an elderly male preponderance and is associated with other autoimmune conditions such as thyroiditis and coeliac disease. The disease is steroid responsive, but many patients experience disease relapse.

**Figure 1:**
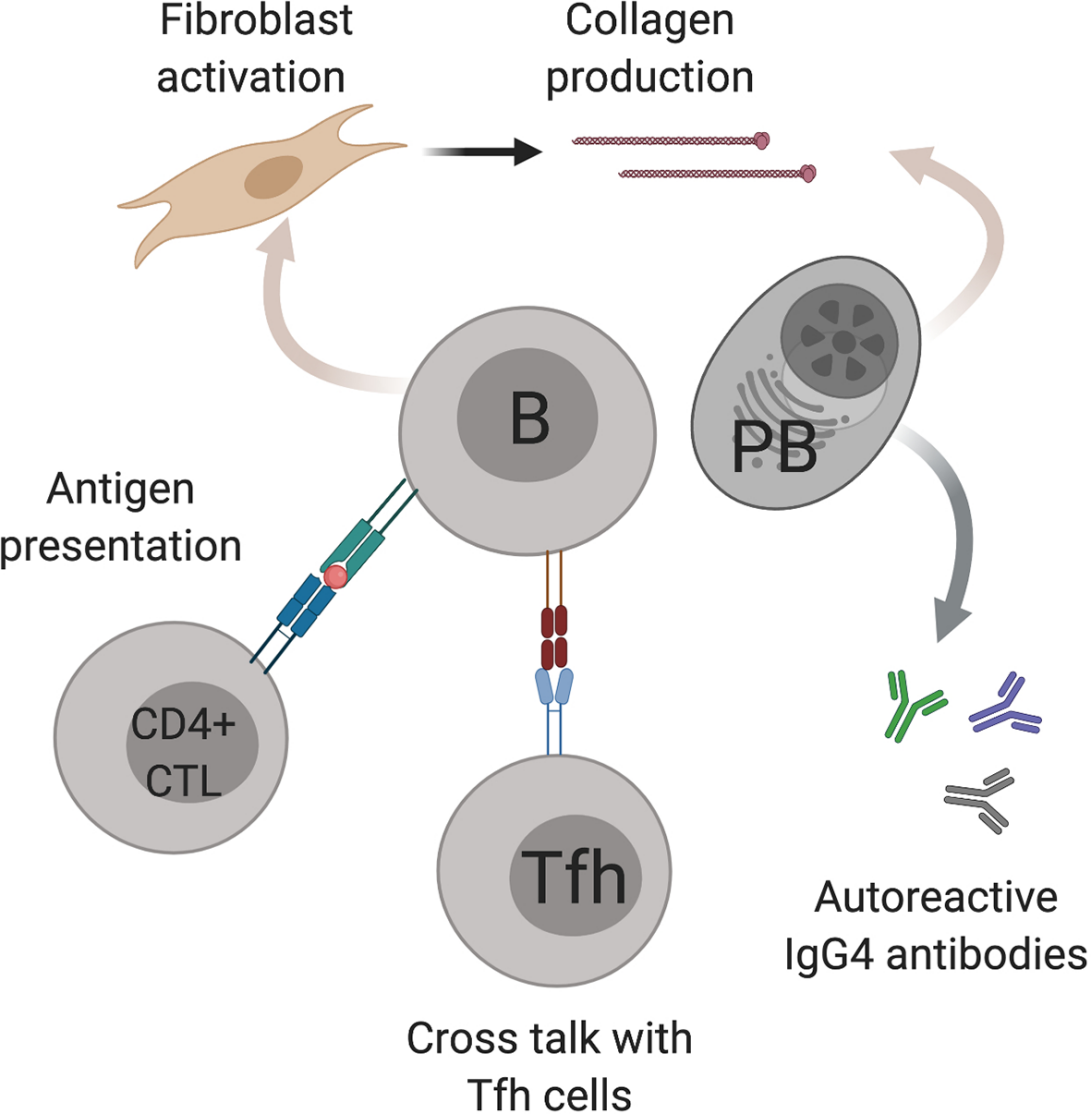
Postulated mechanisms of B cell involvement in IgG4-hepato biliary disease. B cells (B) have several possible mechanisms in IgG4-hepato biliary disease pathogenesis including antigen presentation to cytotoxic CD4 T cells (CD4+ CTL), cross talk with T follicular helper cells (Tfh) and fibroblast activation. IgG4 positive plasmablasts (PB) also contribute to fibrosis as well as producing IgG4 antibodies. Image created using BioRender.com.

B cells play several potential roles in disease pathogenesis including the production of autoreactive antibodies, presentation of antigen to T cells, cytokine/chemokine production and directly contributing to fibrosis. B cell depletion with rituximab has shown efficacy in treating active IgG4-RD as both an induction and maintenance agent, and is commissioned in the UK as a third-line therapy (33). Postulated mechanisms of B cell involvement in IgG4-HBD are summarized in **Figure 1**.

### IgG4 Antibodies in IgG4-HBD

Abundance of circulating and tissue-infiltrating IgG4 antibodies are evident in IgG4-HBD. Elevated serum IgG4 levels are a useful adjunct in disease diagnosis and if elevated they are helpful in disease monitoring and in relapse prediction. Circulating IgG4+ PB also show utility in disease monitoring in those with active disease (34).

IgG4 responses comprise approximately 4% of IgG subclass circulating antibodies in health (35). They have a unique structure and function and can undergo exchange of their antigen binding fragments (Fab) in a process known as Fab arm exchange (36). The resulting heterodimeric antibodies are poor complement binders that are unable to induce the formation of large immune complexes (37, 38). Circulating memory B cells have lower surface expression of complement receptor 2 (CR2, CD21) on IgG4 compared to IgG1 B cells, suggesting IgG4 memory B cells themselves are less responsive to complement signals (39). Hypocomplementemia occurs in IgG4-RD involving the renal, pancreatic and biliary systems, but its role in pathogenesis is not yet clear.

IgG4 B cells are produced in response to repetitive antigenic stimulation (40), developing for example after administration of therapeutic monoclonal antibodies (41). IgG4 responses may be formed as a protective mechanism to dampen immune reactions towards antigens encountered repeatedly [reviewed in (42, 43)]. IgG4 B cells also localize to tissue sites of inflammation (44), supporting that their recruitment may be a local mechanism to contain immune responses. Both type 2 helper T cells (Th2) and their signature interleukins (IL) -4, -10 and -13, and Tfh cells producing IL-21 are associated with IgG4 production (45–52). However, in IgG4-HBD the exact mechanisms triggering and sustaining the predominance of the IgG4 subclass remain elusive.

### Autoantibodies and B Cell Clonality in IgG4-HBD

Excess IgG4 antibodies may be autoreactive to an as yet unidentified antigen. Autoantibodies against a range of different antigens such as galectin-3, prohibin, annexin A11 and laminin 511-E8 have been identified in patients with IgG4-RD (53, 54). However, all are non-specific ubiquitous proteins, expressed with variable frequencies across organs in patients with systemic disease. Indeed, polyclonal expansion of IgG4 autoantibodies has been demonstrated in response to multiple common environmental antigens in IgG4-HBD (55), indicating the predominance of IgG4 responses might be a more generalized phenomenon.

Next generation sequencing data of both circulating IgG4 B cells and PBs shows oligoclonal expansion of B cell receptor clones in IgG4-HBD (56–58). Circulating PB that re-expand on disease relapse have a different clonality to those at disease presentation (58). This suggests alternative epitopes might drive subsequent rounds of inflammation, although it does not rule out a common driving autoantigen. Further studies are required to understand *in vivo*, whether IgG4 B cells are pathogenic themselves or whether other immunological conditions drive and sustain B cell proliferation and IgG4 antibody production.

### Antigen Presentation to Pathogenic T Cells in IgG4-HBD

Expanded B cells and PBs may present antigen to pathogenic subsets of T cells in IgG4-HBD. Expansion of clonally restricted CD4+ SLAMF7+ cytotoxic T cells (CTLs) cells that interact with B cells and produce pro-fibrotic cytokines have been demonstrated in the circulation and inflamed tissue of individuals with IgG4-RD. Depletion of B cells with rituximab leads to profound clinical and radiological improvement in disease activity, paralleled by a decline in PB and CD4 CTLs, implying B cells are necessary to sustain the expanded CTL population (59, 60).

Antigen-experienced B cells can regulate Tfh cell differentiation by priming naïve CD4 T cells and polarizing them towards IL-21-producing Tfh cells that enhance immunoglobulin production in cocultured B cells (61). Tfh are activated and expanded in IgG4-HBD, and play a crucial role in B cell differentiation and GC formation in involved organs (51, 52, 62–66). Circulating activated Tfh cells have been observed to positively correlate with PB number, IgG4 class switch promoting cytokines such as IL-4, serum IgG4 levels, disease activity and organ involvement in IgG4-HBD (51, 52, 62–66). It is plausible that bidirectional signaling between Tfh and B cells might sustain a cycle of IgG4 humoral responses in IgG4-HBD, although this has yet to be shown definitively *in vivo*.

### Fibrogenic B Cells in IgG4-HBD

Storiform fibrosis is an integral histopathological feature of lesions in IgG4-HBD. Indirect evidence that B cells play a role in fibrosis came from histological sections showing B cells in close proximity to fibrotic ducts in IgG4-HBD (67). Further evidence shows *in vitro* B cells derived from individuals with IgG4-RD are able to activate primary pancreatic human fibroblasts to produce collagen (67). PBs rather than naïve or memory B cells were most successful in collagen production when cultured *in vitro* with fibroblasts. Transcriptomic analysis showed genes associated with fibroblast proliferation, including lysyl oxidase homolog 2 *(LOXL2)*, were upregulated and demonstrated direct expression of collagen by PBs themselves (67). B cell depletion with Rituximab reduces clinical disease activity, the Enhanced Liver Fibrosis score (ELF score), a surrogate marker of fibrosis (68), and the number and size of tissue myofibroblasts in IgG4-RD. These observations support a role for B cell depletion in treating those with fibrotic disease and the potential to reverse fibrosis in the longer term.

### The Gut Microbiome in IgG4-HBD

A single microbiome study in IgG4-RD evaluating stool microbiome by metagenomics showed IgG4 patients to have a decreased alpha diversity compared with healthy controls, and expansion of potentially pathogenic and pro inflammatory species such as Th17-inducing strain of *E. lenta* (69). There was potential microbiome-driven skewing of the immune cell population to favor both fibrotic and pre-inflammatory pathways in this disease, with microbial similarities seen to systemic sclerosis (in terms of fibrosis, perhaps sharing the CTLs that shape B cell proliferation and antibody production) and rheumatoid arthritis (in terms of inflammation) but differing to the pathogens observed in inflammatory bowel disease (69). Further studies to understand the relationship between the gut microbiome and B cells in IgG4-HBD as well as its response to B cell depletion therapy will further our understanding of its role in disease pathogenesis.

## Primary Biliary Cholangitis

Primary biliary cholangitis is a chronic cholestatic disease characterized by inflammation of the small intrahepatic bile ducts, resulting in fibrosis and cirrhosis (70). PBC has a female preponderance and is frequently associated with concurrent autoimmune disorders such as Sjögren’s syndrome. Immunosuppressive agents are not effective in PBC treatment, but ursodeoxycholic acid (UDCA) is able to induce disease remission in a proportion of patients.

In PBC both environmental and genetic risk factors contribute to a loss of immune tolerance towards biliary epithelial cells (71). Although T cells have been implicated in disease pathogenesis, B cells are also thought to play an important role. B cells and PCs can be detected in the inflammatory infiltrate surrounding bile ducts in PBC (72). Circulating CD19+ B cells are increased in PBC, and positively correlate with biochemical indices of disease activity including alkaline phosphatase (ALP) levels (73). Genetic studies have identified several risk loci coding for proteins involved in B cell development and function including *CD80*, *CXCR5*, *POU2AF1, SPI, IKZF3*, *ARID3A* and *CD40L* (74–79). Current evidence supports a role for B cells in PBC pathogenesis though autoantibody production, cytokine secretion and reduced regulatory functions.

### Autoantibodies and B Cell Clonality in PBC

Antimitochondrial antibodies (AMA) are detectable in the majority of individuals with PBC and, being highly sensitive and specific, are useful for disease diagnosis (70). Pyruvate dehydrogenase complex - E2 subunit (PDC-E2), a major target of AMA, is aberrantly expressed on biliary epithelial cells in individuals with PBC (80).

Several lines of evidence support that AMAs might be pathogenic in PBC. The frequency of B cells specific to mitochondrial antigens were found to be correlated with increasing stage of PBC in the early inflammatory phase (81). Antibody secreting PBs and PCs isolated from the livers of individuals with PBC were able to produce AMA *ex-vivo* (82) and PCs surrounding inflamed bile ducts on liver biopsies were observed to correlate with AMA titers (72). AMA have also been shown to stimulate macrophages to produce pro-inflammatory cytokines (83) that can directly drive inflammation in PBC.

The association of PBC with AMA and their ability to inhibit energy generation by the pyruvate dehydrogenase complex led to the hypothesis that fatigue in PBC might be ameliorated by B cell depletion. However a phase II randomized controlled trial of treatment with the B cell depleting agent Rituximab in PBC conferred no significant improvement in fatigue (84), despite being associated with reduced AMA titers (84, 85). Further, a minority of PBC patients do not have detectable AMAs, suggesting they might not be pathogenic in of themselves. CD4 T cells with autoreactivity to PDC-E2 are present even in AMA negative persons with PBC, indicating T rather than B cell responses towards PDC-E2 might drive immunopathogenesis (86).

Alternative auto-antibodies can be useful to diagnose individuals with AMA negative PBC (70). The presence of the antinuclear antibodies anti-Sp100 anti-gp210 are associated with a more severe clinical course (87, 88). Other autoantibodies include kelch-like 12 (KLHL12) and hexokinase 1 (HK1) are specific to PBC (89, 90). Studies investigating the clonality of the B cell repertoire in PBC have reported an oligoclonal expansion within the B cell repertoire and have identified disease associated clones shared between different PBC donors (91, 92). This suggests the selection of common autoantigens might drive the altered B cell clonality in PBC, but it is unclear why loss of tolerance towards self-antigen occurs.

As well as specific autoantibodies, a polyclonal IgM response is commonly observed in PBC which is mirrored by infiltrating IgM positive PBs in the portal tracts on liver histology (93–95). T cell expression of CD40-ligand (CD40L) can interact with CD40 on B cells, promoting B cell activation and differentiation. In PBC, methylation of the CD40L promotor is reduced, resulting in increased CD40L expression on CD4 T cells and higher serum IgM levels (96). CD40L has also been identified as a central upstream regulator in PBC by GWAS and microarray analysis (79), supporting that T and B cell communication via the CD40L-CD40 axis has an important role in pathogenesis.

### B Cell Expansion and Activation in PBC

Studies of circulating lymphocytes in PBC have observed increases in CD19+ B cells and activated CD25+ B cells, correlating with disease stage (73, 97). Within the B cell compartment, different B cells subsets seem to be altered including decreased circulating memory B cells and increased naïve B cells and PBs (73, 98). In liver biopsy specimens from individuals with PBC, CD20+ B cells were found in lymphoid follicle like aggregations a distance from portal tracts, or more scantily as part of the lymphoplasmacytic infiltrate surrounding inflamed bile ducts. Plasma cells expressing IgM or IgG were consistently found surrounding bile ducts in a coronal distribution alongside CD4 and CD8 T cells, and thus local interactions between PCs and T cells via cytokine production or direct antigen presentation are possible (72).

Several factors have been identified that might drive B cell expansion in PBC. CXCR5+ Tfh cells, necessary for B cell differentiation, were found to be expanded in the blood and liver of individuals with PBC and *ex-vivo*, enhanced AMA production from autologous B cells in co-culture (73). Furthermore the Tfh associated cytokine IL-21 is increased in the serum and livers of treatment naïve PBC patients, is positively correlated with the percentage of circulating PBs and reduces with successful treatment with UDCA (73). In addition, CD19+ B cells have higher expression of the IL-21 receptor in PBC, suggesting they might be more receptive to IL-21 signaling (73). BAFF, another signal associated with B cell maturation, has been shown to be increased in PBC and positively correlates with AST and bilirubin (99). These observations support that B cell expansion and activation in PBC are driven by signals such as BAFF and IL-21 from Tfh cells.

### Cytokines Regulating Inflammation and Recruitment in PBC

There is evidence that B cells produce cytokines in PBC that might contribute to disease pathogenesis.

*Ex-vivo* CD19+ B cells from PBC patients produced increased inflammatory cytokines interleukin-6 (IL-6) and tumor necrosis factor alpha (TNF-α) compared to CD19+ cells from healthy controls (73). Circulating CXCL10, which can be produced by a variety of cells including B cells, has been observed to be increased in PBC patients and is expressed in portal regions or by hepatocytes in areas of focal necrosis (25, 100). These data support that B cells might contribute the inflammatory milieu in PBC, leading to the perpetuation of inflammation.

Other reports have investigated anti-inflammatory regulatory B cells in PBC. One study found although peripheral B regulatory cells (CD19+CD24^hi^CD38^hi^) were increased in PBC, *ex-vivo* these cells produce lower inhibitory signals such as IL-10 and showed less inhibitory activity towards CD4+ T cells (101). Instead, proinflammatory cytokines such as IL-6 and IL-12 were produced and CD4 T cell differentiation towards a Th1 phenotype occurred (101). However, other studies have not detected differences in circulating B regulatory cells in PBC (73) and a recent analysis of immune cells at the single cell level found a different population of regulatory B cells with a B10 phenotype (high CD24 and IgD expression) were decreased rather than increased in PBC patients (98). Together these results suggest the role of different B cell subsets in PBC pathogenesis is likely to be nuanced, and dependent on the fine tuning of pro- and anti- inflammatory cytokines.

### The Gut Microbiome in PBC

Several studies support that the gut microbiome may play an important role in PBC pathogenesis. AMAs have been found to cross react with several bacterial proteins including *Escherichia coli*, which might initiate their production early in PBC (21, 102–104). Changes in the fecal microbiome may drive the polyclonal IgM response in PBC, which is less diverse in persons with PBC than in healthy counterparts (105–107). Specific bacterial populations in PBC are associated with alterations in bile acids, which are thought to directly contribute to biliary epithelial cell damage (108). Alterations in bile acid composition correlate with serum IgM in PBC both before and after treatment (108). Further work is required to determine causality between these associations.

## Primary Sclerosing Cholangitis

Primary sclerosing cholangitis is a chronic disease characterized by immune mediated damage of the biliary tree resulting in concentric fibrosis and stricturing of the extrahepatic or intrahepatic bile ducts (109). Currently there is no effective treatment. PSC is strongly associated with Inflammatory Bowel Disease (IBD). PSC-IBD subjects have been shown to have a distinct gut microbiome as compared to individuals with IBD alone or healthy controls (110), leading to the hypothesis that loss of immunological tolerance to shared gut and liver antigens might be involved in PSC pathogenesis. The role of B cells in PSC pathogenesis is incompletely understood, but there is evidence that autoantigens towards microbiome sensing proteins and antibody secreting plasma cells might play a role.

### Autoantibodies and B Cell Clonality in PSC

The association of several autoantibodies with PSC and evidence of their potential roles in pathogenesis is accumulating. Anti-neutrophil cytoplasmic antibodies (ANCA) are commonly detected in PSC, but their presence is not specific, and over 10 antigenic targets have been proposed [reviewed in (111)]. In one large European PSC cohort, 80% were positive for ANCA predominantly with a perinuclear staining pattern (p-ANCA) (112). Whether the presence of ANCA antibodies in PSC is protective or detrimental is not yet clear. ANCA specific for proteinase 3 (PR3), detected in 35% of PSC patients in one cohort, was shown to positively correlate with liver transaminase and ALP levels (113). An association between ANCA negative PSC patients and biliary cancer has also been reported (112).

Through the connection of PSC with IBD, common autoantibody targets have been investigated. Autoantibodies of the IgA subclass targeting glycoprotein 2 (GP2) were identified in patients with both PSC and IBD (114, 115). GP2 is not known to be expressed on the biliary epithelium but is present on intestinal microfold (M) cells and plays a role in antigen sensing of the gut microbiome (116, 117). This supports that the gut-liver axis might be key in PSC pathogenesis and loss of tolerance may initially occur in the gut (reviewed in (118)). The presence of anti-GP2 antibodies in individuals with PSC is associated with cholangiocarcinoma, increased mortality and reduced transplant free survival (119, 120), indicating anti-GP2 antibodies might play a pathogenic role. However observational studies report only 31–50% of PSC patients are anti-GP2 positive (120), suggesting other mechanisms are likely to be relevant.

Autoantibodies directed at the biliary epithelium itself have also been investigated. Sera from individuals with PSC has been shown to induce the production of pro-inflammatory cytokines such as IL-6 by biliary epithelial cells (121, 122). Supernatants from liver infiltrating B cells isolated from PSC patients and cultured *ex-vivo* have been shown to react to several proteins in an array assays, including nucleolar protein 3 (NOD3), expressed by cholangiocytes (82). B cell clonality studies also point to the involvement of as yet unidentified auto-antigens, with individuals with PSC exhibiting increased B cell clonality in the liver and gut compared to healthy tissue (123). This study also found significant overlap in expanded B cell clones shared between the gut and liver (123), further supporting the hypothesis that loss of tolerance in the gut is important.

### Liver Infiltrating B Cells in PSC

A single study has addressed the phenotype of infiltrating B cells in PSC livers (123). Although B cells were significantly lower than in livers from PBC patients, PSC specimens had proportionally higher percentages of IgA or IgG positive PBs amongst antibody-secreting cells, and *ex-vivo* were able to secrete IgA and IgG immunoglobulins (82). This supports that PBs might be the predominant local source of autoantibody production in PSC, although further work is necessary to validate this and understand the mechanisms maintaining PB expansion.

Mdr2^-/-^ mice which lack the biliary transport protein Mdr2, demonstrate sclerosing cholangitis and progressive liver fibrosis and have been used to model autoimmune cholestatic diseases including PSC (124). A recent study investigating the role of B cells in the pathogenesis of this model has revealed intrahepatic B cells produce IgG constitutively, the levels of which positively correlated with serum BAFF. BAFF blockade or depletion of CD20+ B cells by monoclonal antibody reduced hepatic fibrosis in this model (125), raising the possibility that similar effects could be seen with B cell depletion in PSC patients. However, as the primary deficit in this model is due to impairment in bile transport rather than alterations in the gut-liver axis, the translation of these findings to PSC may not bare out.

### The Gut Microbiome in PSC

In PSC and PSC-IBD, microbiome association studies in both stool and the intestinal mucosa have identified alterations in the bacterial component of the microbiome (126). Increased relative abundance of *Enterococcus*, *Veilonella* and *Streptococcus* genera have been reported in multiple studies, with *Klebsiella pneumoniae*-harboring microbiomes from patients with PSC/UC being implicated as a driver of Th17 responses in gnotobiotic mouse models (127). Human studies to examine the relationship between the gut microbiome and B cells in PSC will be required to understand whether they are causally linked.

## B Cell Responses to Immunomodulatory Therapies

### Corticosteroids

Corticosteroids are steroid hormones used as first line induction therapy to control inflammation in patients with AIH, IgG4-HBD and autoimmune overlap with cholestatic liver disease, usually leading to both clinical and biochemical improvement (13, 70, 109). On reduction of steroid dose or withdrawal of medication, disease relapse occurs frequently (>80% in AIH and 35-60% in IgG4-HBD) (128, 129). Prednisolone therapy has been shown to decrease activated T cells, inhibit the differentiation of B lymphocytes into PC, reduce IL-10 and IL-21 cytokine levels, and the expression of BLIMP-1 and Bcl6 that regulate PC differentiation in mouse models of autoimmune disease (130). Intra-hepatic B cells and T regulatory cell proliferation is suppressed by prednisolone in adults with AIH (131). Circulating PBs and activated PD1+ Tfh cells are decreased and clonal B cell expansion is reduced within 12 weeks of prednisolone therapy in adults with IgG4-HPB disease (57, 132, 133).

### Ursodeoxycholic Acid

Ursodeoxycholic acid (UDCA) is a secondary bile acid used to treat cholestatic liver diseases. UDCA main mechanisms of actions include a) protection of cholangiocytes against cytotoxicity of hydrophobic bile acids, b) stimulation of hepatobiliary secretion and c) protection of hepatocytes against bile acid induced apoptosis. In patients with PBC UDCA improves liver biochemistry, may delay disease progression to severe fibrosis/cirrhosis, and prolongs transplant free survival. In patients with PSC it improves liver biochemistry and surrogate markers of prognosis (70, 109). In patients with IgG4-HBD it may also improve liver biochemistry but has no known impact on disease progression or survival. Reductions in CD19+ B cells and B cell clonal expansion have been observed after UDCA treatment in patients with PBC (92), although it is not known if this has a direct or indirect relationship to UDCA treatment, and whether the observed alterations in the B cell compartment contribute to disease remission. Similar studies in PSC and IgG4-HBD in response to UDCA have not been done.

### Rituximab

Rituximab is a monoclonal antibody which targets the humoral immune response by inducing B lymphocyte depletion and decreased production of autoantibodies. It has been used to treat individuals intolerant or unresponsive to standard therapy therapies in AIH and IgG4-HBD (28, 114) and in a clinical trial for symptomatic fatigue in PBC (84).

A prospective open label trial (134) and several retrospective observational studies of between 10 and 60 participants support rituximab as an effective agent to treat active IgG4-RD [**Table 2** (68, 134–141)]. Maintenance treatment with further rituximab infusions reduces the risk of IgG4-RD relapse compared with induction therapy alone (141), but this has not been confirmed in randomized trials. Side effects including infusion reactions, hypogammaglobulinemia and infection have been reported (134–136, 139, 140). In IgG4-HBD, rituximab not only depletes CD20+ B cells, but also short-lived CD20- PBs and reduces cytotoxic CD4+ T cells (137, 138). Long-lived CD20- PCs that reside in the bone marrow are also not depleted and might represent a niche of pathogenic cells that drive disease relapse. If disease reactivation occurs expanded PBs of different clonalities are observed (59), indicating B cells with different antigen affinities might be involved in repeated rounds of inflammation. Interestingly, rituximab leads to selective decreases in serum IgG4 levels in both patients with IgG4-RD and in Rheumatoid arthritis where serum IgG4 levels at baseline are normal (134, 144, 145). This suggests short-lived PB are the main contributors to serum IgG4 levels and disease activity is reduced through depletion of these cells.

**Table 2:** Studies of Rituximab for treatment of IgG4-RD.

Study	Study Design	Intervention	Participant number	Treatment population	Adverse events	Efficacy outcome
Khosroshahi et al. (135)	Retrospective single centre cohort	Rituximab x2 1g 15 days apart Repeat dose if disease relapse occurred	10	Active IgG4-RD Steroid refractory	- Asthma flare - HBV reactivation	- Clinical improvement in 90% (n=9) within 1 month. - Steroids discontinued by median 5.3 months. - Recurrence in 20% (n=2) by 6 months. - Complete remission in 40% (n=4) at 6 months.
Hart et al. (136)	Retrospective single centre cohort	Rituximab x4 375 mg/m^2^	12	IgG4-related AIP Steroid refractory or intolerant	- Infusion reaction - Neutropenia - Bronchiolitis obliterans	- Complete remission 83% (n=10) post induction. - Radiological improvement 80% (n=8) by median 4.5 months. - Reduced IgG4-RI and serum IgG4.
Carruthers et al. (134)	Prospective open label trial	Rituximab x2 1g 15 days apart	30	Active IgG4-RD Steroid refractory	- Infection	- Disease response in 97% (n=29) at 6 months. - Primary outcome (reduction IgG4-RI, no disease flare and off steroids) achieved in 77% at 6 months (n=23). - Complete remission in 40% (n=12) at 12 months. - Recurrence in 10% (n=3) by 6 months and 13% (n=4) by 12 months.
Wallace et al. (137)	Retrospective single centre cohort	Rituximab x2 1g 15 days apart	12	Active untreated IgG4-RD	Not reported	- IgG4-RI decreased from 13.8 to 4.4 and plasmablasts decreased from median 6356 cell/mL to 1419 cell/mL between 3 and 6 months after treatment.
Della-Torre et al. (68)	Retrospective single centre cohort	Rituximab x2 1g 15 days apart	10	Active IgG4-RD, no hepatic disease	Not reported	- IgG4-RI decreased from 13.1 to 4.2 and ELF score decreased from 8.3 to 6.3 at 4 months. - Relapse in 20% (n=2) by 13 months.
Wallace et al. (138)	Retrospective single centre cohort	Rituximab x2 1g 15 days apart	60	Active IgG4-RD	Not reported	- Relapse in 37% (n=21) by median 253 days. Relapse incidence 0.39 per person year. - Baseline serum IgG4, IgE and absolute eosinophil levels associated with relapse.
Ebbo et al. (139)	Retrospective multicentre cohort	Rituximab x2 1g 15 days apart or x4 375 mg/m^2^	33	IgG4-RD treated with rituximab	- Infusion reaction - Infection - Hypogammaglobulinemia	- Clinical response in 93.5% of those with symptoms (n=29/31). - Relapse in 42% of responders (n=13/31) by median 19 months. Mean relapse free survival of 30 months. - IgG4-RI over 9 points at baseline was predictive of relapse.
Wallwork et al. (140)	Retrospective single centre cohort	Rituximab x2 1g 15 days apart	26	IgG4-related or idiopathic retroperitoneal fibrosis Steroid refractory	- Infusion reaction - Severe infection	- Symptomatic improvement in 100%. - Radiological improvement in 88%.
Campochiaro et al. (141)	Retrospective single centre cohort	Rituximab x2 1g 15 days apart induction Group 1 = further dose on relapse Group 2 = maintenance treatment every 6 months	14	Active IgG4-RD	- Infusion reaction - Infection	- Clinical response in 100% after 1 month. - Complete remission in 36% after 6 months. - Disease remission in 57% after 6 months. - Free relapse rate significantly lower in group 2 (n=0/7, on maintenance) compared to group 1 (n=5/7, no maintenance).

IgG4-RD, IgG4-related disease; AIP, autoimmune pancreatitis; IgG4-RI, IgG4 responder index (142); ELF score, enhanced liver fibrosis score (143); disease remission, IgG4-RI score under 3 points off steroid therapy; complete remission, IgG4-RI score under 3 points on treatment; partial response, decrease in IgG4-RI score of over 2 points but total score remains over 3 (142).

A single center open label trial of rituximab in 6 AIH patients and a retrospective cohort of 22 AIH patients in France between 2007 and 2017 demonstrated significant improvements in serum IgG and liver transaminases sustained for up to 24 months after treatment and reported no significant adverse events (9, 10). Compared to IgG4-RD, AIH involves a more cell-mediated immune process, however rituximab has been used successfully in other diseases with a similar mechanism such as multiple sclerosis, indirectly altering cell-mediated responses (146). Mouse models have shown that B lymphocyte depletion impairs CD4+ T cell activation in response to pathogen challenges, which may in part explain the effect of rituximab in AIH. In paired liver biopsies of AIH before and after rituximab therapy, inflammation grade which correlated with CD4 regulatory T cells, improved with treatment (10). This suggests B cell depletion in AIH might work therapeutically through an indirect reduction in liver infiltrating CD4 T cells.

Rituximab has been shown to reduce auto-antibody production (AMA, IgM) and biochemical values (ALP) in a small number PBC patients unresponsive to standard therapy with UDCA (85, 147). A randomized trial of rituximab in 57 early stage PBC patients on UDCA did not significantly improve fatigue (primary end point), but those in the treatment arm had significantly lower ALP levels 3 months after infusion (84). Assessment of B cell function in PBC patients after rituximab demonstrated that depletion of B cells influences the induction, maintenance, and activation of both B and T cells. Transient decreases in memory B cell and T cell frequencies and an increase in T regulatory cells are observed. This is associated with increased in mRNA levels of forkhead box P3 (FoxP3) and tissue growth factor-beta (TGF-β) and a decreased TNF-α in CD4 T cells after B cell depletion treatment (147).

## Novel B Cell Therapeutic Targets

**Figure 2:**
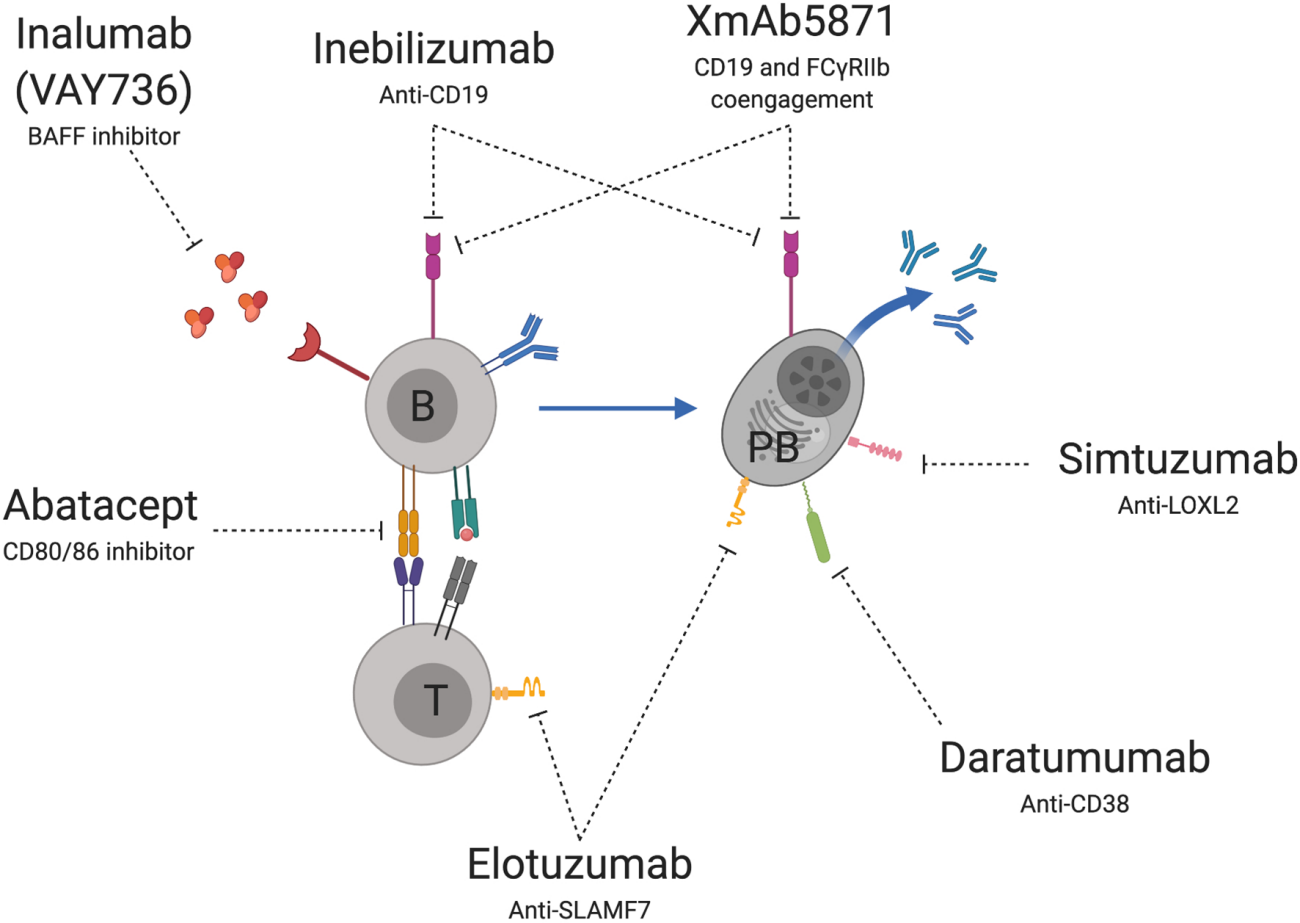
Novel B cell therapeutic targets for immune-mediated liver diseases. Treatments under development or evaluation include agents that deplete or disrupt B cells (B) or plasmablast (PB) signaling through CD-19, disruption of PB activation through CD-38, inhibition of PB mediated fibrosis through LOX-2 and inhibition of SLAM-F7 on PB and T cells (T). Other targets prevent B cell signaling through soluble mediators such as BAFF and receptor mediated T cell (T) co-activation through CD80/86. Image created using BioRender.com.

Several novel therapies are under investigation for immune-mediated liver diseases that directly or indirectly target the B cell lineage (**Figure 2**). These have various mechanisms of action including B cell depletion, inhibition of direct B cell signaling through cell-cell interactions and inhibition of B cell signaling through cytokine production.

Agents that directly target B cells include monoclonal antibodies specific for the CD19 receptor. Treatment leads to depletion of CD19 expressing cells including B cells and PBs and has shown efficacy in treating other autoimmune disorders where B cells play a role in pathogenesis including systemic sclerosis (148), multiple sclerosis (149), and neuromyelitis optica (150). A randomized placebo-controlled trial of Inebilizumab, a monoclonal antibody specific for CD19, is currently underway in patients with active IgG4-RD (151).

Other monoclonal antibodies that directly target B cells or PBs inhibit signaling through specific surface receptor targets. These include LOXL2, which plays a role in fibrogenesis in PSC and IgG4-RD (67), SLAMF7 which is expressed highly on PBs and CTLs in IgG4-RD (59, 60) and CD38 which is expressed on expanded PBs in IgG4-RD (34, 58, 132, 152). Simtuzumab, a monoclonal antibody specific to LOXL2, has been evaluated in 234 PSC patients for its ability to reverse fibrosis. However it did not achieve a reduction in hepatic collagen content on liver biopsy or improve Ishak fibrosis stage (153). It had not been evaluated other autoimmune liver diseases. Elotuzumab, a monoclonal antibody specific to SLAMF7 and Daratumumab, a monoclonal antibody specific to CD38 are also available [reviewed in (154)] but have not been evaluated for therapy of autoimmune liver disease.

Other monoclonal antibodies alter B cell or PB surface receptor co-engagement with receptors on other cells. These include interruption of T cell activation by antigen presenting B cells through blocking CD80/86 – CD28 signaling by Abatacept, and suppression of B cell responses through co-engagement of the Fc-gamma receptor IIb with CD19 by XmAb5871 (155–157). Abatacept has shown efficacy in other B cell mediated autoimmune conditions such as Rheumatoid arthritis (158). Disappointingly, an open label trial of Abatacept in 16 PBC patients who had not responded to UDCA therapy reported that only a single patient met the primary endpoint of a reduction in ALP of over 40% from baseline and there were no significant reductions in serum IgM (159). However, clinical trials of Abatacept in IgG4-RD and AIH are ongoing and have not yet reported (160, 161). Results of XmAb5871 in autoimmune liver diseases have been more promising. XmAb5871 was evaluated in a prospective open label clinical trial in 20 individuals with active IgG4-RD (162). The primary outcome, improvement in IgG4 disease responder index by over 2 points by day 169 after therapy, was achieved in 80% of participants. During follow up, pneumonia, chronic lymphocytic leukemia and chronic inflammatory demyelinating polyneuropathy occurred certain individuals, but may not have been directly related to therapy (162, 163).

B cells and PBs are also indirectly targeted by several therapeutic agents. For example, B cell activating factor receptor (BAFF) inhibitors such as Inalumab (VAY736), prevent BAFF signaling and indirectly lead to B cell depletion. Inalumab has recently shown safety and efficacy in a phase II trial in individuals with primary Sjögren’s syndrome, a B cell mediated autoimmune disease involving exocrine glands (164) and has been used successfully to induce remission in 2 reported cases of difficult to treat AIH (165). A randomized controlled trial assessing Inalumab (VAY736) for the treatment of AIH is currently recruiting (166).

Finally, novel therapeutic agents indirectly targeting B cell signaling through cytokine production are also available. For example NI-0801 is a monoclonal directed towards CXCL10, which is produced by B cells and is increased in the serum of PBC patients (25, 100). NI-0801 has been trialed in 29 PBC patients that were unresponsive to UDCA but unfortunately was not efficacious at reducing ALP levels (167).

Currently the majority of novel B cell therapies are in early-stage clinical trials and their associated risk profiles are being elucidated. In the case of Rituximab, which is used for the treatment of several autoimmune conditions and lymphoproliferative disorders, the risks of treatment include reactivation of latent viral illness, hypogammaglobulinemia and in impaired B cell reconstitution, which can result in recurrent or reactivation of infection (168, 169). The extent to which these risks might occur with other B ell depleting or suppressing therapies in the specific context of treating autoimmune liver disease requires investigation. Despite these potential risks, overall novel B cell therapies hold great promise to improve the treatment options for autoimmune liver diseases going forward.

## Current Research Gaps and Potential Developments in the Field

Current data support that B cells play a role in autoimmune liver disease pathogenesis, through autoantibody production, interaction with T cells via antigen presentation or cytokine signaling. Evidence that B cell depletion with rituximab can induce disease remission has led to support for its use for treatment refractory cases of IgG4-RD and AIH (33, 170). Several other treatments targeting B cells are under development for the treatment of immune mediated liver diseases, for example monoclonal antibodies targeting CD19 or BAFF. Some of these agents are in phase I therapeutic trials, which if successful could increase the armory of therapeutic agents for the treatment of autoimmune liver conditions (160–162, 166).

Due to the relative rarity of autoimmune liver disease, the evaluation of novel therapies with sufficient power to detect efficacy will need to be done collaboratively on a national and international scale. Inclusive trials that evaluate efficacy in a group of rare conditions with similar pathogenesis is one mechanism to recruit sufficient patients, an approach that has been taken with other immune mediated rare diseases such as the vasculitidies (171, 172). In the UK, nationally recruiting cohort studies for AIH [AIH-UK (173)], IgG4-RD [IgG4-systemic disease (174)], PBC [PBC-UK (175)] and PSC [PSC-UK (176)] provide an existing network for clinicians and scientists working collaboratively on autoimmune liver diseases, that could be harnessed for national therapeutic clinical trials.

The holistic patient experience living with autoimmune liver disease covering the spectrum of symptoms, diagnosis, treatment and management pathways should also be considered when evaluating B cell targeting therapies. Recent data from the UK-AIH national cohort study indicate quality of life is significantly impaired in AIH patients, as is particularly associated with steroid use (177). A recently published systematic review of patient reported outcomes in studies of PBC and PSC concluded that although the use of patient reported outcomes has increased over time, many are nonspecific and unvalidated (178). This underscores the importance in developing and evaluating relevant patient reported outcomes in clinical trials of novel therapeutics alongside clinical efficacy signals.

Finally, studies evaluating immunotherapies should focus on in depth evaluation of the immunological response to therapy both in the peripheral blood and if possible, at the site of disease. Recent data has shown liver fine needle aspirate (FNA) to safe in assessing the hepatic immunological milieu and has highlighted differences between immune responses in the blood and the liver (179). Studies evaluating the immunological response to B cell targeting therapies in autoimmune liver diseases should consider employing FNA to understand the local response to treatment, and in turn develop more targeted therapies towards B cells within the liver itself.

## Author Contributions

TC and EC jointly wrote and edited the manuscript. All authors contributed to the article and approved the submitted version.

## Funding

EC is supported by the National Institute of Health Research (NIHR) Biomedical Research Centre, based at Oxford University Hospitals Trust and Oxfordshire Health Service Research Committee (OHSRC) as part of Oxford Hospitals Charity, Oxford. TC receives funding from a Wellcome Trust Training Fellowship for Clinicians [211042/Z/18/Z]. The views expressed in this article are those of the authors and not necessarily those of the NHS, the NIHR, or the Department of Health.

## Conflict of Interest

EC is on the advisory board for Viela Bio for IgG4-realted disease.

The remaining author declares that the research was conducted in the absence of any commercial or financial relationships that could be construed as a potential conflict of interest.
